# Monitoring of Lake Ice Phenology Changes in Bosten Lake Based on Bayesian Change Detection Algorithm and Passive Microwave Remote Sensing (PMRS) Data

**DOI:** 10.3390/s23249852

**Published:** 2023-12-15

**Authors:** Yimuran Kuluwan, Yusufujiang Rusuli, Mireguli Ainiwaer

**Affiliations:** 1 Laboratory of Basin Information Integration and Ecological Security, College of Geography and Tourism, Xinjiang Normal University, Urumqi 830054, China; emran717@163.com (Y.K.); mihary@xjnu.edu.cn (M.A.); 2 Key Laboratory of Arid Lake Environment and Resources, Urumqi 830054, China

**Keywords:** PMRS, Bayesian change detection, lake ice, freeze–thaw process, BL

## Abstract

Lake ice phenology (LIP), hiding information about lake energy and material exchange, serves as an important indicator of climate change. Utilizing an efficient technique to swiftly extract lake ice information is crucial in the field of lake ice research. The Bayesian ensemble change detection (BECD) algorithm stands out as a powerful tool, requiring no threshold compared to other algorithms and, instead, utilizing the probability of abrupt changes to detect positions. This method is predominantly employed by automatically extracting change points from time series data, showcasing its efficiency and accuracy, especially in revealing phenological and seasonal characteristics. This paper focuses on Bosten Lake (BL) and employs PMRS data in conjunction with the Bayesian change detection algorithm. It introduces an automated method for extracting LIP information based on the Bayesian change detection algorithm. In this study, the BECD algorithm was employed to extract lake ice phenology information from passive microwave remote sensing data on Bosten Lake. The reliability of the passive microwave remote sensing data was further investigated through cross-validation with MOD10A1 data. Additionally, the Mann–Kendall non-parametric test was applied to analyze the trends in lake ice phenology changes in Bosten Lake. Spatial variations were examined using MOD09GQ data. The results indicate: (1) The Bayesian change detection algorithm (BCDA), in conjunction with PMRS data, offers a high level of accuracy and reliability in extracting the lake ice freezing and thawing processes. It accurately captures the phenological parameters of BL’s ice. (2) The average start date of lake ice freezing is in mid-December, lasting for about three months, and the start date of ice thawing is usually in mid-March. The freezing duration (FD) of lake ice is relatively short, shortening each year, while the thawing speed is faster. The stability of the lake ice complete ice cover duration is poor, averaging 84 days. (3) The dynamic evolution of BL ice is rapid and regionally distinct, with the lake center, southwest, and southeast regions being the earliest areas for ice formation and thawing, while the northwest coastal and Huang Shui Gou areas experience later ice formation. (4) Since 1978, BL’s ice has exhibited noticeable trends: the onset of freezing, the commencement of thawing, complete thawing, and full freezing have progressively advanced in regard to dates. The periods of full ice coverage, ice presence, thawing, and freezing have all shown a tendency toward shorter durations. This study introduces an innovative method for LIP extraction, opening up new prospects for the study of lake ecosystem and strategy formulation, which is worthy of further exploration and application in other lakes and regions.

## 1. Introduction

According to the latest report from the Intergovernmental Panel on Climate Change (IPCC) in 2021, the average global temperature increased by 1.1 °C compared to the average temperature during the period of 1850–1900, covering the years 2011 to 2020. More concerning is the prediction that, in the near future (i.e., 2021 to 2040), there is a high likelihood of surpassing the critical threshold of 1.5 °C. This escalation would further exacerbate the ongoing global warming trend [1]. Despite covering only about 2% of the Earth’s land surface, lakes play a critical role in the Earth’s ecosystem [2]. The water circulation processes of lakes not only have a significant impact on local climate change, but also have a close relationship with local human activities, particularly in arid regions [3]. Lake ice, as a significant component of the cryosphere, is being affected by climate change. Its phenological processes, including freeze-up start (FUS), freeze-up end (FUE), break-up start (BUS), and break-up end (BUE), influence the hydrological processes and aquatic ecosystems of lakes. Therefore, the rapid and accurate extraction of lake ice information using remote sensing technology is crucial for understanding regional climate change, lake ecosystem response, and the development of effective ecological protection strategies.

In high-latitude regions, the seasonal changes in lake ice also exhibit periodic phenological patterns. Over the past few decades, the duration of lake ice cover in the Northern Hemisphere has been continuously decreasing, and this trend is expected to become even more severe in the context of future warming [4]. Related research has shown that climate change has a direct and significant impact on natural ecosystems on Earth [5]. Specifically, the impact of climate change on water circulation in arid regions has been a recent focus of study [6]. Currently, LIP research primarily focuses on the extraction of temporal and attribute parameters. While temporal parameters can be obtained through field observations, this method is often time consuming and labor intensive. Moreover, for many high-altitude lakes, there is a lack of on-site observation data regarding LIP characteristics. As a result, researchers are increasingly turning to remote sensing technology and modeling methods to study LIP. For example, Stephen [7] used QuikSCAT microwave data to extract the LIP period of the Great Bear Lake and Great Slave Lake in northern Canada from 2000 to 2006, based on significant differences in the backscatter coefficients between the water and ice. Using AVHRR data, they observed two lakes in the Baltic region from 1980 to 2012 and used a threshold method to extract lake ice phenological changes [8]. High-resolution radiometer AVHRR data were employed to analyze the freeze–thaw process of Qinghai Lake and explore the relationship between lake ice thickness and temperature using observational data [9]. Cai et al. analyzed the freeze–thaw dates and lake ice coverage time of over 50 lakes on the Qinghai-Tibet Plateau, from 2000 to 2017, using MODIS data and further investigated the spatiotemporal differences and reasons for the lake ice phenological changes [10]. Wu et al. used MODIS and AMSR-E data to identify the ice conditions of Nam Co Lake and found that MODIS was the most accurate in determining the start of ice thawing [11], while AMSR-E was more accurate in monitoring the time to complete freeze-up and complete thawing [11].

Remote sensing technology enables us to acquire essential information about lake ice morphology and spatial distribution, while modeling methods can help systematically extract LIP information. Qiu et al. empirically demonstrated the effectiveness of the threshold method for LIP using PMRS data, but due to insufficient data resolution, it does not adequately support the study of LIP in small and medium-sized lakes [12]. Ruan et al. used PMRS data with a 25 km resolution to study the LIP of Nam Co Lake using a mixed pixel decomposition method, but they could not distinguish between lake shore and lake water pixels, resulting in some errors [13]. Existing LIP studies have paid relatively little attention to the changes in LIP in Xinjiang. Qin et al. used MODIS data to study the LIP changes in Sai Li Mu Lake in Xinjiang [14]. Aierken et al. used optical remote sensing data to study and explore the characteristics of LIP changes in large lakes in Xinjiang, but the time series was short and the method was not as comprehensive as traditional methods, making it less accurate for studying long-term LIP characteristics [15]. Cai et al. used a threshold method to calculate the LIP changes of 23 lakes in Xinjiang and concluded that changes in area and mineralization could also affect the LIP changes of lakes [10]. Therefore, introducing a more efficient method for calculating LIP in the context of climate change is of great significance for this research. Unlike optical remote sensing methods, microwave remote sensing: (1) is not affected by cloud or rain weather conditions; (2) has a higher (at least once in a day) revisit frequency, allowing for the acquisition of daily lake surface brightness temperature information; (3) has a long time series from 1987 to the present, SSMR sensors data are available from 1978 to 1987, SSMI sensors data are available from 1987 to 2008, and SSMIS sensors data are available from 2000 to the present [12]; (4) by leveraging the significant temperature differences between lake ice, open water and land, the freezing and thawing dates of lakes can be extracted.

In past research, various time series algorithms have been developed to utilize the temporal dimension of satellite data, and this remains a dynamic field of research. The advantage of time series analysis in depicting landscape dynamics and disturbances is evident, but the availability of many alternative algorithms highlights a potential issue: no single algorithm is always applicable to all scenarios. Recently, a study evaluating seven common methods emphasized this issue and found that interference detected between algorithms was nearly zero at the pixel level [16]. One approach to alleviate this dilemma is to abandon the single best algorithm paradigm and turn to ensemble modeling. This ensemble algorithm was originally reported by Zhao et al. for the Bayesian estimation of mutations, seasonality, and trends [17]. The first evaluation of the algorithm was a case study monitoring the wetland vegetation dynamic [18]. Conceptually, the Bayesian Estimator of Abrupt change, Seasonal change, and Trend (BEAST) combines many individual weak models into a better model through Bayesian model averaging. BEAST is rigorously formalized, and its key equations are manageable in analysis. In practical applications, BEAST can estimate the probability of change point occurrence, detect not only major disturbances but also low-level disturbances, and reveal complex nonlinear trend dynamics, all of which are challenging for single best model algorithms. BEAST is not only suitable for remote sensing data, but also for other environmental, ecological, or socioeconomic time series data.

This paper is based on the BECD algorithm for the extraction of lake ice phenological parameters from microwave remote sensing data. This method is formulated within a Bayesian hierarchical modeling framework and is implemented using mixed MCMC sampling chains to analyze the seasonal, trend, and change point components of high-density brightness temperature data. Optical remote sensing data are used to validate and evaluate the feasibility of this algorithm in this study. The goal is to explore the trends and features of Bosten LIP since 1978 and propose a more efficient and rapid method for extracting LIP.

## 2. Study Area

**Figure 1:**
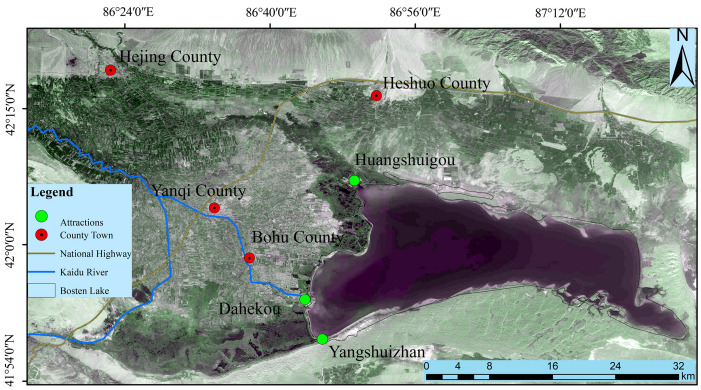
Overview map of the study area.

Figure 1 displays an overview of the study area, depicting the geographical features of Bosten Lake. BL, the largest inland freshwater lake in China, is situated in the lowest depression of the Yanqi Basin in Xinjiang [19]. The terrain exhibits a northwest-to-southeast slope, and the lake serves as both the terminal point of the Kaidu River and the source of the Kongque River. With a water surface area of 1646 square kilometers, an average depth of 9 m, and a maximum depth of 17 m, BL is located between 86°40′ to 87°25′ E and 41°56′ to 42°14′ N. It stretches approximately 55 km from east to west and 2 km from north to south, with an elevation of 1048 m. It ranks as the largest inland freshwater flow-through lake in China. The region belongs to a temperate continental arid climate zone. This climate type is characterized by four distinct seasons, with hot and dry summers, cold and dry winters, an annual average temperature below 10 °C, and an annual precipitation of less than 400 mm. In such a climate, water resources are relatively scarce, leading to frequent occurrences of drought and water shortages. Additionally, the pronounced diurnal temperature variation and arid climate conditions pose challenges to the local ecosystem and biodiversity. In summary, BL is a geographically unique lake located in a region with an arid climate in Xinjiang. The lake ecosystem is influenced by climate and water resource variations.

## 3. Materials and Methods

### 3.1. Microwave Remote Sensing Data

This paper utilizes the CETB product provided by the National Snow and Ice Data Center (NSIDC, https://nsidc.org/data/, accessed on 20 March 2023) to extract LIP information for BL from data collected by the SMMR, SSM/I, and SSMIS sensors. The SMMR sensor was carried on the Nimbus-7 satellite and featured five frequency bands (6.6 GHz, 10.7 GHz, 18.0 GHz, 21.0 GHz, and 37.0 GHz), with a temporal resolution of 1 day and spatial resolutions of 25 km, 3.125 km, 12.5 km, and 6.25 km, respectively. The SSM/I and SSMIS sensors were mounted on satellites in the Defense Meteorological Satellite Program (DMSP) series (F 8, F 10, F 11, F 13, F 14, F 15, F 16, F 17, F 18, and F 19) and included four frequency bands (19.35 GHz, 22.2 GHz, 37.0 GHz, and 85.5 GHz, with SSMIS replacing 85.5 GHz with a 91.655 GHz channel). This study utilized data with a spatial range covering BL and a temporal range from October 1978 to December 2022, with a spatial resolution of 3.125 km and a temporal resolution of 1 day.

### 3.2. Optical Remote Sensing Data

The Moderate Resolution Imaging Spectroradiometer (MODIS) is sourced from the Satellite Observing Center at the National Aeronautics and Space Administration (https://modis.gsfc.nasa.gov/, accessed on 23 March 2023). The MODIS sensor is mounted on both the Terra and Aqua satellites in NASA’s Earth Observing System, featuring a total of 36 bands, with spatial resolutions of 250 m, 500 m, and 1000 m. This study made use of the MOD09GQ dataset, obtained from the Terra satellite, and the MYD09GQ dataset, acquired from the Aqua satellite. Both datasets feature a spatial resolution of 250 m and a temporal resolution of one day. For the MOD09GQ dataset, data from 1 August 2000 to 31 December 2022 were utilized. As for the MYD09GQ dataset, data from 1 August 2002 to 31 December 2022 were employed. The data in this study are used for the visual verification of the accuracy of the BECD in extracting LIP processes from PMRS data and capturing the spatial variations in lake ice for the given lakes.

Additionally, the daily snow product from MODIS is sourced from the National Snow and Ice Data Center in the United States (https://nsidc.org/data/, accessed on 23 March 2023). This paper incorporated the MOD10A1 snow product dataset from the Terra satellite and MYD10A1 dataset from the Aqua satellite, both offering a temporal resolution of one day and a spatial resolution of 500 m. Data spanning from 2000 to 2022 were chosen for validation of the parameters, such as the onset of freezing, complete freezing, the onset of thaw, and complete thaw, extracted by our research algorithm. The results of this study indicate that the accuracy of the land cover classification with MOD10A1 data ranges from 87.5% to 94%. Hence, this dataset serves as valuable research support and validation for our study [20]. The purpose of this data in the present study is to cross-validate the accuracy of the LIP information extracted from the PMRS data for the period 2000 to 2022.

## 4. Research Methods and Validation

### 4.1. Nearest Neighbor Algorithm

During each sensor detection of the lake surface, slight differences in the coordinates of each pixel occur due to the sensor’s position. To select the image data with the largest detected lake area during each transit, the image closest to the center coordinates of the lake is often chosen. To identify the central location of the lake, one can select the appropriate image by calculating the distance to the nearest point from the lake’s center. This calculation involves buffering a rectangular area of 3.125 km × 3.125 km on the image centered on the lake’s coordinates to ensure that the image pixel coordinates encompass the lake’s center point. This selection method ensures that the chosen images cover the central region of the lake to obtain the maximum lake area information. In the specific implementation process, image selection is based on finding the point (X min, Y min) closest to the coordinates (X, Y) from the center point (O). This ensures that the selected image is in the closest proximity to the lake’s central position. This strategy is commonly used in remote sensing data analysis to obtain the most relevant and representative image data for further research on the lake’s characteristics and changes. 

### 4.2. Bayesian Change Detection Algorithm

The BECD is a powerful tool for analyzing time series data and detecting changes over time. This method is based on Bayesian inference and, by sampling the posterior distribution of the number and locations of change points, it provides confidence estimates for the change points. It offers researchers precise change point locations and the probability of change point occurrences. The BECD method introduces Bayesian statistics in addressing change point detection problems, making it more flexible. This method considers the statistical properties of the data and utilizes prior information to infer the location and intensity of the change points.

In time series data, change point detection can reveal abrupt changes, shifts in trends, or anomalous events, providing valuable information for decision making and further analysis. Compared to traditional change point detection methods, the BECD method is better suited to handle data noise and uncertainty.

### 4.3. Bayesian Ensemble Change Detection Algorithm

In mathematical terms, the BECD method decomposes a time series Y(t) into four components: trend (T), seasonal variation (S), change points (θt), and noise (ε). These components are combined into the time series model as follows:

Y(t) = T(θ) + S(θ) + ε
(1)

where ε represents Gaussian random errors N(0, δ^2^), with an unknown variance δ^2^. T and S represent the basic components of the trend and seasonal variation, while change points are represented by parameters θ t and θ s. Specifically, θ t and θ s denote the number and location of the change points in the trend and seasonal components.

Introducing Equation (1) allows for the decomposition of the brightness temperature data of BL, enabling us to explore the following inquiries:

1. First, how many change points occurred, and when did they occur? Are these change points indicatives of trends or seasonal variations [21]? The concept of change points represents the trajectories of changes in the brightness temperature due to seasonality and trends. This algorithm offers broader and more inclusive assumptions compared to other algorithms.
2. What are the potential trends? Trends are not limited to linear changes but may also involve complex nonlinear trajectories with multiple change points within a day. Detecting change points with high fidelity is essential for understanding BL’s ice phenology.
3. What are potential seasonal change points? Seasonal change points also encompass trend change points, and they are related to the ice phenology of BL. Seasonal change points may not necessarily include trend change points but can reflect the driving factors of ice phenology changes.

### 4.4. Data Analysis Methods

**Figure 2:**
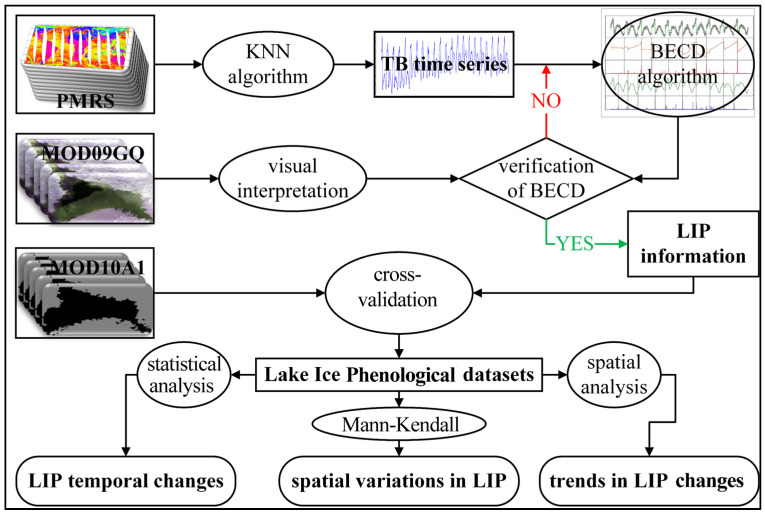
Research framework diagram.

In this study, we aimed to validate the accuracy and reliability of the BECD algorithm on passive microwave remote sensing data, with a spatial resolution of 3.125 km. We employed the correlation coefficient (r), root mean square error (RMSE), and mean absolute error (MAE) for the evaluation, cross-validating the results with lake ice information extracted from the MOD10A1 dataset spanning from 2000 to 2022 [22,23]. To gain a more comprehensive understanding of the lake ice phenological changes in Lake Baotou from 1978 to 2022, we conducted in-depth analysis using the Mann–Kendall non-parametric test to examine the trends and directions of these changes [24,25]. This series of methods and analyses contribute to ensuring the accuracy and reliability of our research findings regarding lake ice variations. All methods and details of the data processing procedures are outlined in Figure 2.

## 5. Results

### 5.1. Extraction and Accuracy Evaluation of Lake Ice Freeze–Thaw Process Information

**Figure 3:**
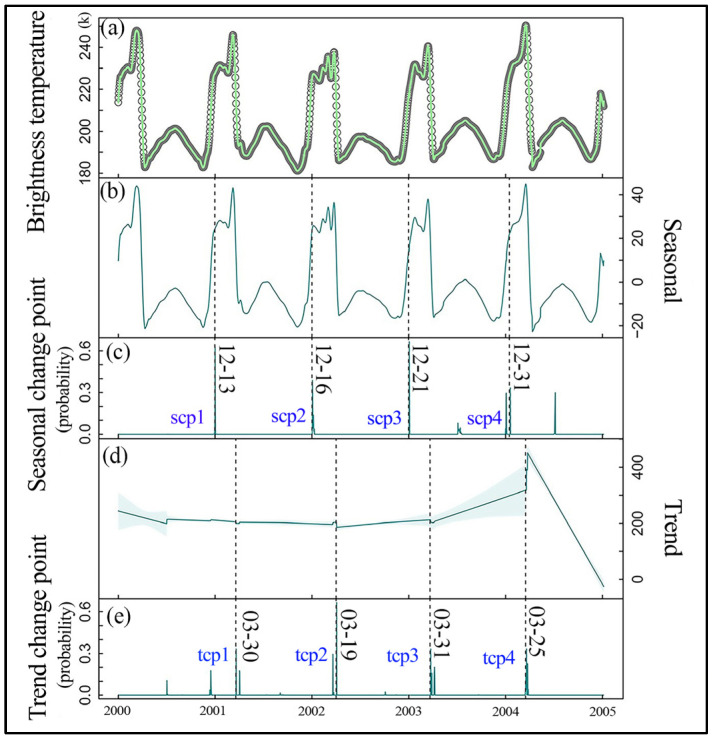
Bayesian ensemble detection decomposition and change point detection graph for brightness temperature in Bosten Lake from 2000 to 2005. In the figure: (**a**) the original data represents the brightness temperature (K); (**b**) the algorithm decomposes the original data to extract the seasonality; (**c**) the algorithm identifies the seasonal change points, such as tcp1, along with the occurrence probabilities; (**d**) the algorithm decomposes the original data to reveal the trend; (**e**) the algorithm detects the trend change points, such as scp1, along with the occurrence probabilities.

A time series of brightness temperature values at a resolution of 3.125 km using PMRS data was utilized, and the BECD algorithm was applied to this data to obtain the range of lake ice freeze–thaw dates from 1978 to 2022. Taking the example of change detection in the time series brightness temperature values from 2000 to 2005, an illustration of the decomposition of the time series of the brightness temperature values related to lake ice is presented (refer to Figure 3). In this figure, the BECD algorithm detected five seasonal change points (Figure 3c) and five trend change points (Figure 3e), with higher probabilities associated with their occurrences. This indicates the probability of abrupt changes in the brightness temperature values in BL and suggests that the times at which these changes are highly likely to occur are closely related to the phenology of lake ice, based on differences in the brightness temperature values between open water and ice. It is important to note that Figure 3 is an example, showing only a small portion of the results on the decomposition of the time series of the brightness temperature values related to lake ice. The BECD algorithm is capable of conducting a more comprehensive analysis of the entire time series, providing more detailed and accurate information about LIP changes. 

**Figure 4:**
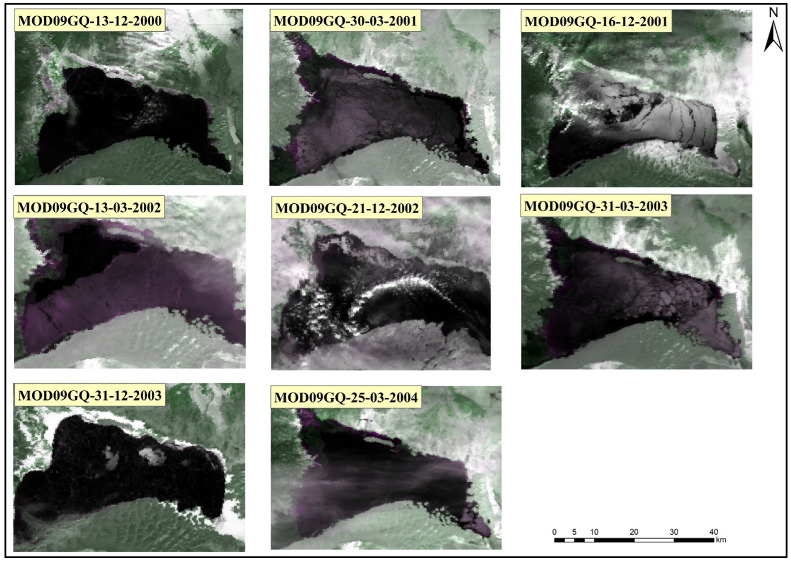
RGB remote sensing images from MOD09GQ during the freeze–thaw period from 2000 to 2004.

The seasonal and trend change point probabilities were estimated using a Bayesian Model Averaging (BMA) strategy, and the threshold for mutation probability can be manually controlled to determine the change point locations based on prior knowledge. To validate the reliability of the Bayesian ensemble change detection algorithm, the time series extracted using optical remote sensing data was used to verify the timeframe of the Bayesian change detection. Since Landsat data is synthesized every eight days and it is challenging to obtain daily imagery, MOD09GQ data was used as a substitute. Figure 4 shows the remote sensing images corresponding to the detected change point dates using the BECD algorithm.

From Figure 4, it can be observed that ten change points were detected through both seasonal and trend signals (Figure 3), with an average of two changes occurring each year. First, a trend signal captured a change in the brightness temperature values in December 2000, which is highly likely to be related to BL’s ice phenology. Further examination of Figure 4 through visual interpretation indicated that BL entered the initial freezing stage on 13 December 2000.

During the period from 2000 to 2005, the dates of BL’s ice changes obtained by the BECD algorithm were all within the ice phenology’s freezing and thawing process. This indicates that the BECD algorithm accurately captures the freezing and thawing processes of BL’s ice. This has significant implications for the study of lake ice freezing and thawing. The times of the change points extracted by the BECD algorithm, along with the remote sensing images obtained based on this algorithm, allow for a more in-depth understanding of the temporal characteristics and phenology of BL’s ice. This provides a reliable method and data support for the detection and study of lake ice changes.

**Figure 5:**
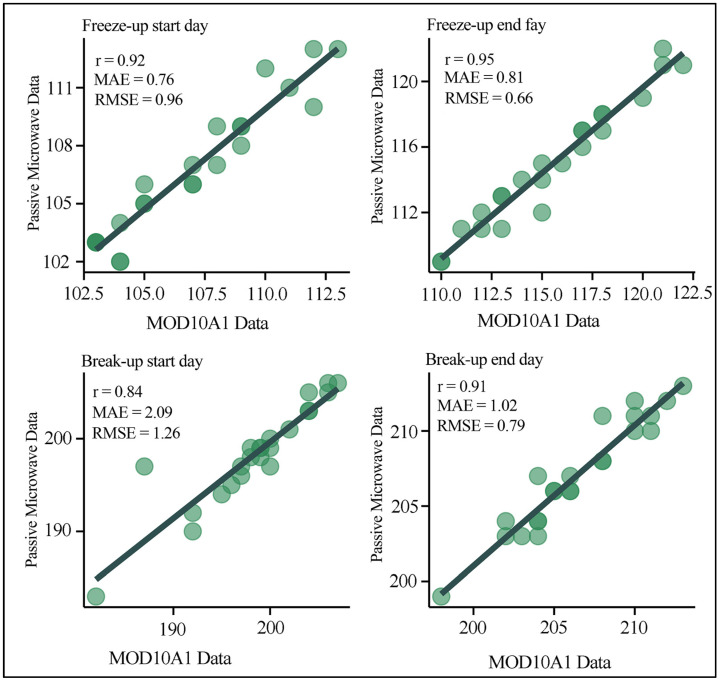
Cross-validation on the ice phenology of Bosten Lake derived from PMRS data and MOD10A1. In this figure, the *X* and *Y* axis represent the extracted dates of lake ice phenology from MOD10A1 data and passive microwave remote sensing data, respectively. The phenological information is derived from every 1 September to 31 August in the next year. Moreover, “r” indicates the correlation coefficient, “MAE” represents the mean absolute error, and “RMSE” represents the root mean square error.

In order to validate the suitability of PMRS data with a spatial resolution of 3.125 km for extracting LIP information, this study conducted cross-validation using 250 m resolution MOD10A1 data from 2000 to 2022. The LIP information extracted from the PMRS data during the same period was utilized for comparison. Based on Figure 5, it is evident that whether it is the start of freezing, complete freezing, the start of thawing, or complete thawing dates, the frozen–thawed dates extracted from the MOD10A1 data show a high correlation with those obtained using the BECD algorithm and PMRS data. In general, the frozen dates extracted from the PMRS data are earlier than those from the MOD10A1 data, and thawing occurs later.

The RMSE for the start of freezing date is 0.968 days, and the MAE is 0.746 days (Figure 5), indicating that the PMRS data extracted the start of freezing date for BL earlier from 2000 to 2022 compared to the MOD10A1 data. This is because the MOD10A1 data were continuously affected by cloudy weather when obtaining the complete freezing date, leading to a delay in extracting the start of freezing date from the MOD10A1 data. The lowest MAE and RMSE values were found for the complete freezing date, with values of 0.81 days and 0.664 days, and an R-squared value of 0.952 (Figure 5). This suggests that due to the lower spatial resolution of the PMRS data, there are limitations to obtaining details accurately. Therefore, there is a slight tendency for the judgment of the complete freezing date to be slightly earlier than the actual date. The start of freezing date (Figure 5) had a maximum MAE of 1.261 and an RMSE of 2.09, indicating that the start of ice melting in BL, as observed in the PMRS data, appears later than in the MOD10A1 data. For the PMRS data, even small ice cover on the lake surface can be reflected by the brightness temperature, but for smaller areas of melting, it may not be accurately represented. In addition, the orbital swath intervals in the PMRS data are likely to contribute to the delay in the start of melting date. The MAE value for the complete melting date is 0.79, and the RMSE is 1.025. Similarly, due to issues with cloud cover in the MOD10A1 data and spatial resolution in the PMRS data, there is a delay in obtaining the complete melting date.

### 5.2. LIP Temporal Changes

**Figure 6:**
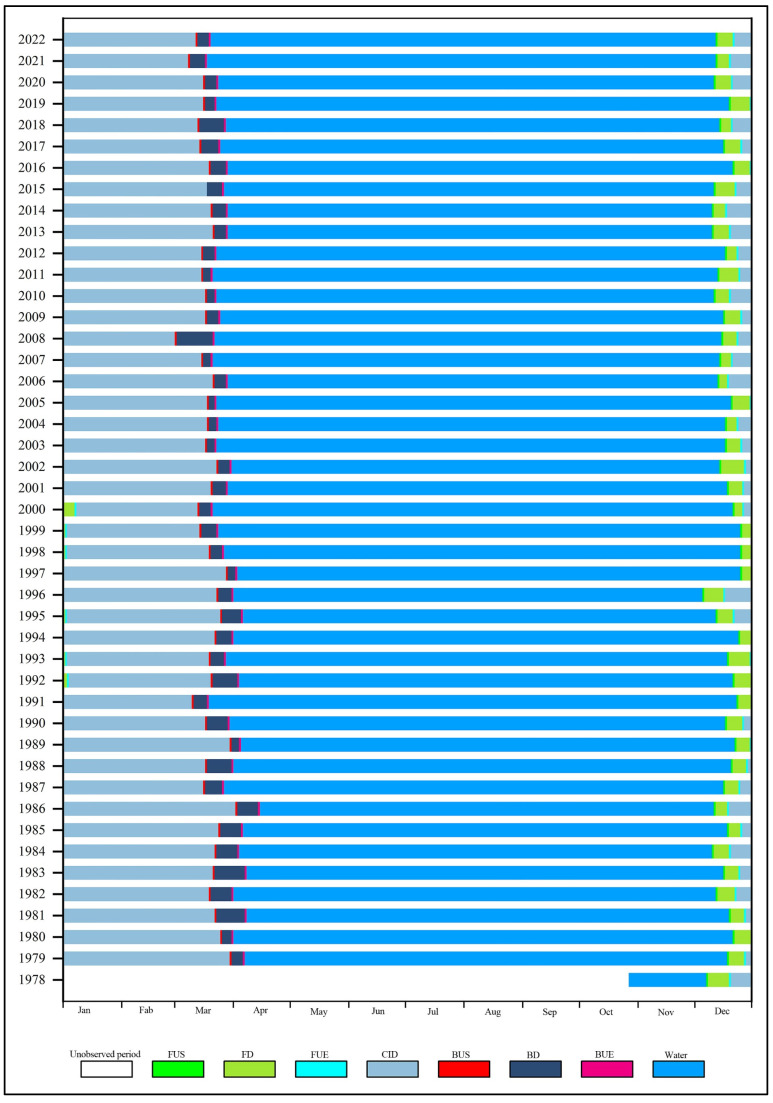
Characteristics of lake ice phenology in Bosten Lake during 1978–2022.

Based on the data presented in Figure 6 and Table 1 which summarizes the ice phenology data extracted from microwave remote sensing of BL, a comprehensive understanding of the ice phenology changes in BL from 1978 to 2022 and the duration of each ice season were obtained. Since the PMRS data began in October 1978, on 28 October 1978, it is categorized as the first year in the 1978/1979 period.

BL typically experiences the start of freezing around mid-December, with an average start date of 108.5 days, which is approximately 17 December. The earliest recorded start date was on 6 December 1996, and the latest was on 2 January 1998. The range of start dates spans 27 days, showing a tendency towards earlier freezing.

The complete freezing of the lake usually occurs in late December, with an average date of around 25 December. The earliest complete freezing date was on 26 December 1996, and the latest was on 8 January 2000. The range of complete freezing dates spans 22 days, indicating a trend towards earlier complete freezing.

The FD represents the duration from the start of freezing to complete freezing, with an average of approximately 8 days. The shortest recorded FD was 4 days in 2006, while the longest was 12 days in 1978, showing a range of 8 days and a trend of shorter FDs.

The start of melting in BL typically occurs around 19 March, with an average date of 201.1 days. The earliest recorded start of melting was on 2 March 2007, while the latest was on 4 April 1985, showing a 33-day difference. The average complete melting date is 210.1 days, occurring on 28 March. The latest complete melting occurred on 14 April 1985, while the earliest was on 17 March 2021, with a range of 29 days. The melting period is an indicator of the local warming rate, with the shortest melting period recorded at 4 days in 2002 and the longest at 23 days in March 2008, indicating a fast-melting rate. 

The CID for BL represents the difference in days between the complete freezing date and the start of melting date. The shortest CID was 71 days in the 2007/2008 period, while the longest was 112 days in the 1985/1986 period, with an average of about 84 days and a range of 41 days. The shortest ED was 81 days in the 1990/1991 period, while the longest was 112 days in the 1985/1986 period, with a range of 39 days. The average ED was 94.13 days, indicating relatively low stability in BL’s ice cover over the past 44 years. 

### 5.3. Spatial Variations in LIP

**Figure 7:**
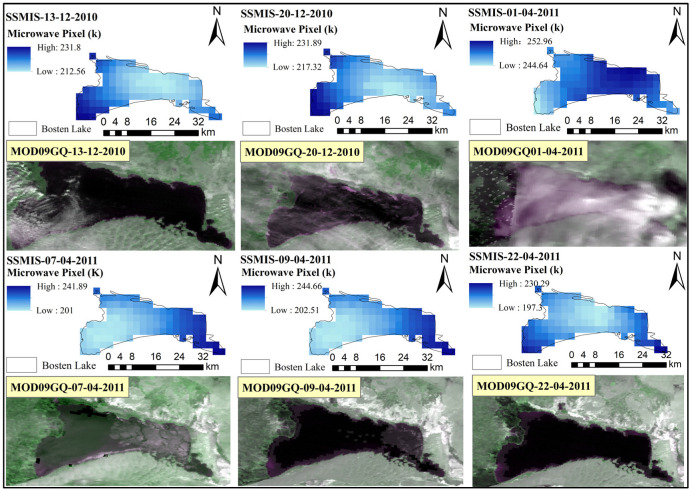
Spatial distribution of lake ice phenology in Bosten Lake (2010–2011).

BL covers a large water surface area, and the dynamic evolution of its ice reflects the differences in the water depth. The formation and melting of lake ice usually start from the shoreline, with shallow water areas freezing earlier compared to deeper regions [26]. Figure 7 presents a study on the dynamic evolution of BL’s ice based on microwave and optical remote sensing images, showing the brightness temperature differences in BL’s ice.

On 13 December 2010, BL entered a freezing state, with the lowest brightness temperature value of 212.56 K in the central area of the lake. The highest brightness temperature value was 231.8 K in the southwest part of the lake. Based on the differences in the brightness temperature values across the lake, it can be determined that BL’s ice on 13 December 2010 was primarily located in the southwestern area of the lake.

On 20 December 2010, the brightness temperature values of BL’s microwave pixels changed, with the lowest value increasing from 212.56 K on the 13th to 217.32 K. Similarly, the high-value microwave pixels expanded from the southwestern and northwestern areas towards the center, indicating ice spreading towards the center of BL.

On 1 April 2011, BL’s microwave pixel brightness temperature values reached a minimum of 244.64 K. Compared to the values in the previous two periods, the brightness temperature values on 1 April 2011 significantly increased and exceeded the previous highest values. This clearly demonstrates that BL was in a fully frozen state.

Based on the maximum and minimum values of the microwave remote sensing pixels on 7 April 2011 (241.98 K and 201 K, respectively), it can be seen that BL was in the melting stage on that day. The main concentration of melting was in the southeast area of the lake, and there were fewer ice pixels.

On 9 April 2011, the high-value microwave remote sensing pixels were compared to those from 1 April 2011. It was observed that on that day, BL had fewer ice pixels, mainly concentrated in the southeast corner of the lake. This indicates that BL completed its ice melting in just 9 days.

On 22 April 2011, the microwave pixel values had a minimum of 197.3 K and a maximum of 203.29 K, which were lower than the highest brightness temperature values from the previous five periods. This suggests that BL was completely ice free on 22 April 2011.

### 5.4. Trends in LIP Changes

**Figure 8:**
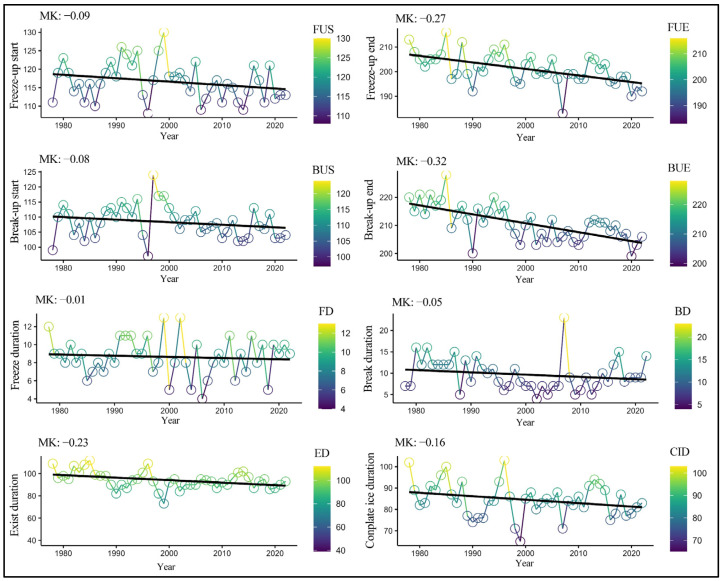
Mann–Kendall trend test graph on Bosten Lake ice phenology from 1978 to 2022. The MK values in the figure represent the annual trend changes for each parameter, measured in units per day. For parameters FUS, FUE, BUS, and BUE, a negative MK value indicates an advanced state. For parameters FD, BD, ED, and CID, a negative MK value signifies a shortened state.

Using the Mann–Kendall non-parametric test method [27] on the microwave remote sensing data extracted for BL’s ice phenology changes, Figure 8 illustrates the trends in BL’s ice phenology, including the dates on the beginning of freeze (FUS), complete freeze (FUE), start of thaw (BUS), and full thaw (BUE), from 1978 to 2022. To further enhance the study of BL’s ice phenology, the trends in additional parameters, such as the break duration (BD), freezing duration (FD), complete ice duration (CID), and exist duration (ED), were analyzed. BL’s ice phenology has shown significant changes since 1978.

The start of freeze date has exhibited a noticeable trend of occurring earlier, with an average advancement of 0.9 days every 10 years. Over the 44-year period, the start of freeze date has advanced by 3.6 days. From Figure 8, it can be observed that BL’s start of freeze date is advancing, with significant fluctuations between 1990 and 2000. 

The start of thaw date has advanced, on average, by 2.7 days every 10 years, totaling an advancement of 10.8 days. This represents an overall trend of earlier thaw dates, although there were noticeable fluctuations between 1990 and 2000. The complete freeze date showed a trend of delay over the 44-year period, with significant fluctuation.

The FD has exhibited a stable trend over the 44-year period, shortening by 0.1 day every 10 years, resulting in a total reduction of 0.4 days.

The trend chart for the start of thaw date shows an overall tendency of occurring earlier, with an advancement of 0.8 days every 10 years, totaling an advancement of 3.2 days over the 44-year period. There were noticeable fluctuations between 1990 and 2000 in the thaw dates.

The trend for the complete thaw date, as shown in the graph, indicates a significant advancement, with an average of 3.2 days every 10 years and a total advancement of 14.8 days over the 44-year period. The fluctuations in the complete thaw date are more pronounced between 1985 and 1995, showing a delay in the complete thaw date.

In the context of ice phenology, the duration of lake ice represents the stability of the ice [28]. The trend analysis of the CID, extracted from microwave remote sensing of BL, shows that the average FD is 84.56 days. The shortest CID occurred in 1999, while the longest occurred in 1996. Over the 44-year period, the CID exhibited frequent fluctuations, mainly shortening, with an average reduction of 1.6 days every 10 years, resulting in a total reduction of 7.04 days and indicating a reduction in the ice stability.

The ED for BL has also shown a consistent shortening trend over the past 44 years, with an average reduction of 2.3 days every 10 years, totaling a reduction of 10.12 days. This further underscores the decreasing ice stability in BL. Both the FD and thaw period can reflect the local climate change status. The FD advanced by 0.5 days every decade, and the thaw period exhibited an advancement rate of 0.1 day/year. The CID shortened by an average of 1.6 days every 10 years, while the ED reduced by 2.3 days every 10 years. 

## 6. Discussion

LIP exhibits seasonal changes [23]. This study employs the BECD algorithm to decompose brightness temperature values from passive microwave remote sensing data, seasonally and trend wise. Combined with the MOD10A1 dataset, the research explores the freezing and thawing processes of BL from 1978 to 2022. The study reveals trends of earlier start of freezing and complete freezing dates for BL. Similarly, the start of thaw date and complete thaw date also exhibit earlier changes. The duration of complete ice cover and ice presence significantly shortens, aligning with previous studies, such as those by Aiken Tursun [15].

The use of time series data has not only unveiled changes in the target object, but has also driven algorithmic development in the field [29]. This study attempts to experimentally demonstrate the effectiveness of the BECD algorithm in detecting mutations, seasonality, and trends. BEAST, as a Bayesian method, separates seasonal and periodic signals from time series data, detects mutations in these signals, and is particularly useful for trend analysis and change point detection in phenological studies [17]. Differentiating it from previous research, this study utilizes the BECD algorithm to better capture the seasonal and trend characteristics of LIP using lake brightness temperature values.

The rapid growth in the use of satellite time series data to reveal landscape changes has spurred algorithm development [29,30]. This progress has led to many new products on ecosystem dynamics, yet it has also opened new research gaps [12]. Many time series methods have been introduced to applications in remote sensing or other disciplines [31]. Many of them are developed under various names, such as trend analysis, seasonal decomposition, breakpoint or breakpoint analysis, signal segmentation, regime shift detection, anomaly detection, and structural change [31,32,33,34]. BEAST provides a universal approximator for any arbitrarily complex trends. In contrast, most existing methods only export linear or piecewise linear trends [35]. The true driving factors behind ecosystem dynamics are unlikely to be purely linear or piecewise linear trends, but rather complex and nonlinear. For example, it is known that plant succession stages largely follow a nonlinear recovery trajectory [36]. Long-term climate trends have been confirmed to have intrinsic nonlinearity [37]. Due to its better approximation capability, BEAST is more likely to find these truly nonlinear trends than existing methods. Improved trend fitting can also help in breakpoint detection, as errors in fitted trends can be translated into errors in breakpoint detection.

The BECD algorithm is effective at detecting dynamic changes in time series data, but it cannot determine the driving mechanisms behind these changes. Seasonal change points often represent certain phenological changes, while trend change points indicate transitions in other dynamics [38]. Whether the rapid freezing and thawing changes in lake ice or long-term trends are related to factors such as temperature, lake mineralization, or precipitation remains unexplored. The BECD algorithm alone cannot fully provide research support for the driving factors in studying LIP. To investigate the driving mechanisms of LIP, integrating the BECD algorithm with other algorithms and auxiliary information is necessary. This integration holds promise for gaining insights into the driving factors behind the changes in LIP of BL from 1978 to 2022. The BECD algorithm can rapidly extract change points from lake brightness temperature values, providing information on LIP. In today’s rapidly changing climate, this method is particularly worthy of utilization in other lakes, such as those in the typically arid region of Xinjiang, China. Combining it with other algorithms can further explore the driving factors. It is essential to note that when studying the LIP characteristics using passive microwave remote sensing data with a resolution of 3.125 km, it is advisable to choose large lakes to avoid mixed pixels that could affect the accuracy of LIP information.

## 7. Conclusions

The study, based on the Bayesian ensemble change detection algorithm, has derived lake ice phenology parameters for Bosten lake from 1978 to 2022 using passive microwave remote sensing data. By employing correlation coefficient analysis, root mean square error, mean absolute error, and the Mann–Kendall non-parametric test, the study analyzed the feasibility of passive microwave remote sensing data and investigated the temporal characteristics and spatial trends of lake ice phenology. The following conclusions have been drawn:

1. The Bayesian ensemble change detection algorithm accurately captured the freezing and thawing dates of Bosten Lake’s lake ice. Through cross-validation with lake ice information extracted from passive microwave remote sensing data on Bosten Lake from 2000 to 2022 and lake ice information obtained from MOD10A1, the minimum correlation coefficient was 0.845. This indicates that the Bayesian ensemble change detection algorithm aligns with the requirements of this study when applied to passive microwave remote sensing data with a spatial resolution of 3.125 km.
2. The lake ice phenology for BL displays distinct trends. The dates for the onset of freezing and complete freezing are being delayed, while the dates for the onset of thawing and complete thawing are occurring earlier. The freezing duration has been shrinking. On average, the freeze-up start date takes place in mid-December, with the freeze-up end date typically occurring by the end of December. The break-up duration usually starts in early March and is typically complete by the end of March. These trends suggest a relatively rapid increase in local temperatures and a decrease in the stability of the lake ice. The shorter freezing duration reflects a faster decrease in the temperature.
3. The dynamic evolution of Bosten Lake ice reveals differences in the water depth across the lake, and the characteristics of ice formation and thawing starting from the lake’s shores and expanding inwards. Freezing usually initiates from the southwest part of the lake, extending towards the central and southeastern regions. As spring temperatures rise, the lake ice melts, and the ice layer thins until it completely thaws.
4. The lake ice phenology for Bosten Lake exhibits significant trend changes. The freeze-up start date is occurring earlier, with an average advancement of 0.9 days per decade. The freeze-up start date is also happening earlier, advancing by an average of 2.7 days per decade. The break-up end date is significantly advancing, with an average advancement of 3.2 days per decade. However, the break-up start date is displaying a delayed trend. The duration and stability of the lake ice are significantly decreasing, indicating the lake’s sensitivity to climate change.

In the study of lake ice phenology, it is essential to explore the driving factors of lake ice phenology. While the powerful tool, the Bayesian ensemble change detection algorithm, can accurately capture lake ice phenological information, it cannot determine the driving factors of the lake ice. To identify the reasons for rapid and slow changes in lake ice, it is necessary to combine the Bayesian ensemble change detection algorithm with other algorithms and data to investigate the driving factors of lake ice.

## Figures and Tables

**Table 1:** Statistics on the Bosten Lake ice characterization information.

Numerical Value	FUS	FD	FUE	CID	BUS	BD	BUE
Average	108.30	8.36	116.60	94.13	201.10	9.66	210.10
Range	27	9	22	39	33	8	41
Annual amplitude	−0.09	−0.01	−0.27	−0.23	−0.08	−0.05	−0.32

## Data Availability

Data are contained within the article.
